# Supplementary material for: Venomix: a simple bioinformatic pipeline for identifying and characterizing toxin gene candidates from transcriptomic data
Source: PeerJ. 2018 Jul 31;6:e5361. doi: 10.7717/peerj.5361 (PMC6074769; doi:10.7717/peerj.5361)
Supplement: Supplemental Information 3 [file peerj-06-5361-s003.gz › FinalOutput_E-6/KappaPI-theraphotoxin-Hs1a_80/finaltree.pdf]

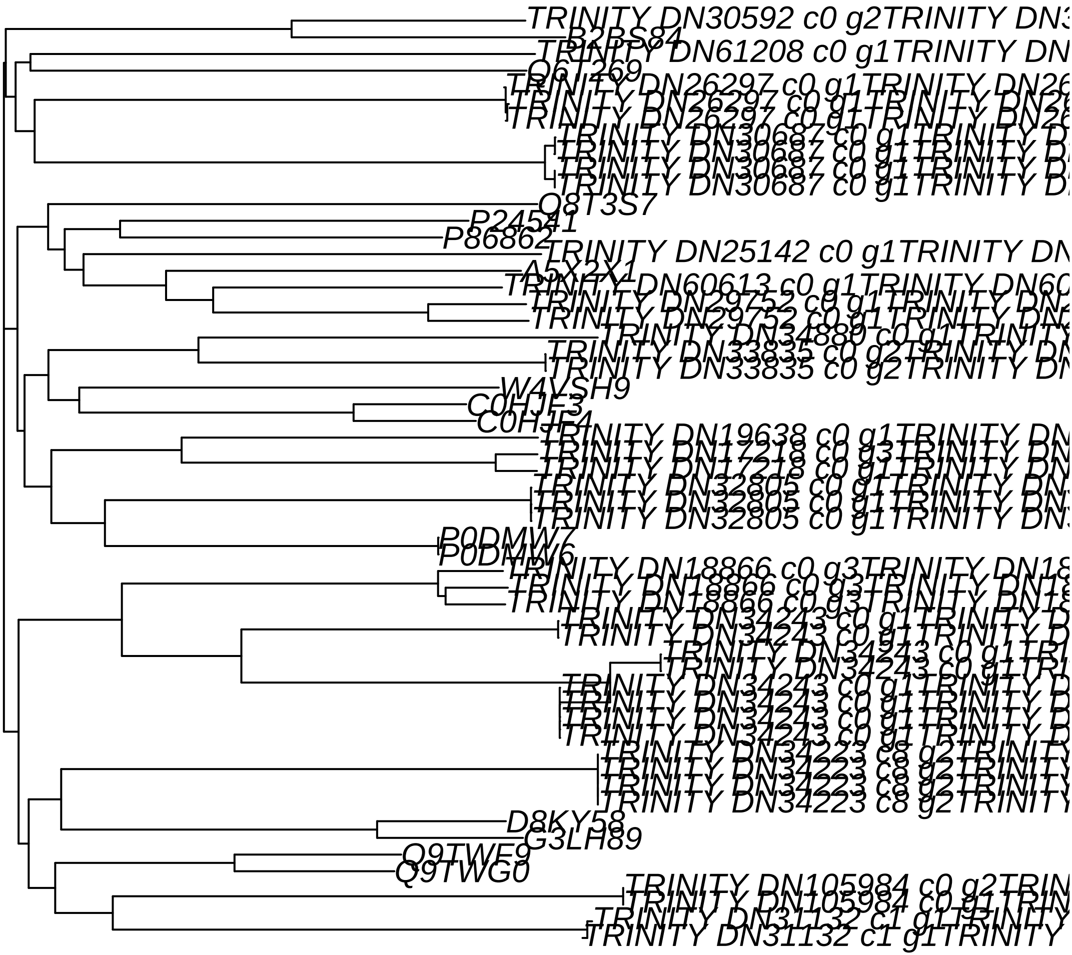
TRINITY DN30592 c0 g2TRINITY DN3
B2BS84
TRINITY DN61208 c0 g1TRINITY DN
Q6T269
TRINITY DN26297 c0 g1TRINITY DN26
TRINITY DN26297 c0 g1TRINITY DN26
TRINITY DN26297 c0 g1TRINITY DN26
TRINITY DN30687 c0 g1TRINITY D
TRINITY DN30687 c0 g1TRINITY D
TRINITY DN30687 c0 g1TRINITY D
TRINITY DN30687 c0 g1TRINITY D
Q8T3S7
P24541
P86862
TRINITY DN25142 c0 g1TRINITY DN
A5X2X1
TRINITY DN60613 c0 g1TRINITY DN60
TRINITY DN29752 c0 g1TRINITY DN2
TRINITY DN29752 c0 g1TRINITY DN2
TRINITY DN34880 c0 g1TRINITY
TRINITY DN33835 c0 g2TRINITY DN
TRINITY DN33835 c0 g2TRINITY DN
W4VSH9
C0HJE3
C0HJE4
TRINITY DN19638 c0 g1TRINITY DN
TRINITY DN17218 c0 g3TRINITY DN
TRINITY DN17218 c0 g1TRINITY DN
TRINITY DN32805 c0 g1TRINITY DN3
TRINITY DN32805 c0 g1TRINITY DN3
TRINITY DN32805 c0 g1TRINITY DN3
P0DMW7
P0DMW6
TRINITY DN18866 c0 g3TRINITY DN18
TRINITY DN18866 c0 g3TRINITY DN18
TRINITY DN18866 c0 g3TRINITY DN18
TRINITY DN34243 c0 g1TRINITY D
TRINITY DN34243 c0 g1TRINITY D
TRINITY DN34243 c0 g1TRI
TRINITY DN34243 c0 g1TRI
TRINITY DN34243 c0 g1TRINITY D
TRINITY DN34243 c0 g1TRINITY D
TRINITY DN34243 c0 g1TRINITY D
TRINITY DN34243 c0 g1TRINITY D
TRINITY DN34223 c8 g2TRINITY
TRINITY DN34223 c8 g2TRINITY
TRINITY DN34223 c8 g2TRINITY
TRINITY DN34223 c8 g2TRINITY
D8KY58
G3LH89
Q9TWF9
Q9TWG0
TRINITY DN105984 c0 g2TRIN
TRINITY DN105984 c0 g1TRIN
TRINITY DN31132 c1 g1TRINITY
TRINITY DN31132 c1 g1TRINITY
